# *Candida* in the ICU, Risk Management and Patient Safety

**DOI:** 10.3390/microorganisms14061200

**Published:** 2026-05-26

**Authors:** Miquel Nolla-Salas, Jordi Ibañez-Nolla

**Affiliations:** 1 Unió Catalana d’Hospitals, 08009 Barcelona, Spain; mnollasalas@gmail.com; 2 Blanquerna School of Health Sciences, Universitat Ramon Llull, 08025 Barcelona, Spain

**Keywords:** candidiasis, intensive care unit, immunoparalysis, multiple organ dysfunction syndrome, *Candida* colonization, antifungal therapy

## Abstract

Endogenous candidiasis remains an underrecognized yet clinically relevant complication in non-neutropenic critically ill patients. This study examines *Candida spp.* infections in the intensive care unit (ICU) within a patient-safety and risk-management framework, focusing on the identification of patients at highest risk and the development of an early diagnostic and therapeutic strategy. The target population comprises long-stay ICU patients requiring prolonged mechanical ventilation who develop multiple organ dysfunction syndrome (MODS) associated with immunoparalysis, typically reflected by a Sequential Organ Failure Assessment (SOFA) score ≥ 5. In this population, *Candida spp.* colonization may evolve into multifocal candidiasis and subsequently invasive or disseminated disease. Notably, candidemia often represents a late manifestation and therefore lacks sensitivity as an early diagnostic marker. Drawing on a series of clinical investigations conducted from 1978 to the early 2000s, the authors developed a standardized diagnostic–therapeutic algorithm based on systematic surveillance cultures, identification of multifocal *Candida spp.* colonization, and early initiation of antifungal therapy. Implementation of this strategy, together with progressive individualization of antifungal treatment, was associated with a marked reduction in attributable mortality related to candidiasis in ICU patients. These findings support the concept of *Candida spp.* infection as a sentinel indicator of systemic immune dysfunction and physiological fragility in critical illness. Integrating risk-based surveillance with early targeted therapy may substantially improve outcomes and reinforce patient-safety strategies in the ICU.

## 1. Introduction

Given the recent publications on *Candida* in the Intensive Care Unit (ICU), particularly those addressing the experiences of patients with prolonged ICU stays during the COVID-19 pandemic [1,2], which have led to the development of several expert consensus statements [3,4,5], it has been deemed necessary to conduct an analysis of the clinical experience in the management of critically ill patients in whom *Candida* was isolated during their ICU stay.

**Figure 1:**
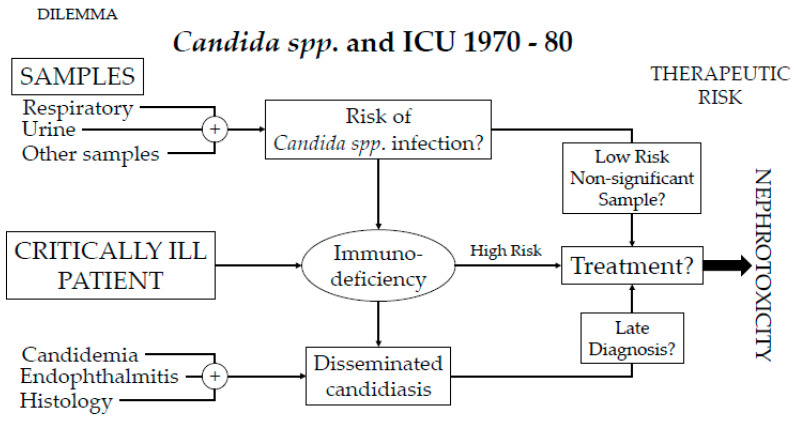
Clinical dilemma posed in 1978: Should positive *Candida* cultures obtained from samples considered non-significant (i.e., from non-sterile sites) be taken into account? In immunocompromised patients, such as those with multiple organ dysfunction syndrome (MODS), do these findings indicate an increased risk of candidemia? When *Candida* is identified in clinically significant samples, is antifungal therapy being initiated too late to improve patient outcomes? At the same time, the potential for antifungal-related adverse events must be considered. Depending on the agent used, treatment may pose additional risks to the patient, such as nephrotoxicity associated with amphotericin B or hepatotoxicity related to azoles. The arrows indicate that, based on the test results—whether or not they are significant—and the patient’s condition, the patient is classified as being at high or low risk for candidiasis and whether or not treatment is required.

This review begins by revisiting a prospective study conducted between 1978 and 1982 [6,7], in which the evolution of cellular immunity and the occurrence of *Candida* infection were analyzed in patients with specific conditions associated with prolonged ICU stays and multiple organ failure (MOF). ICU-acquired candidiasis was regarded as a patient safety concern and, consequently, a risk management issue, closely associated with the aggressive therapeutic interventions applied in this patient population (Figure 1).

Given that patient safety and risk management are intrinsically linked to quality of care, the problem was approached using quality management tools focused on continuous improvement. Based on the results of the initial study, which demonstrated an association between the degree of immunodeficiency and the occurrence of candidiasis, as well as between mortality, immunodeficiency, and candidiasis, it was decided to implement the Deming Cycle methodology. This approach led to the development of a quality circle model aimed at facilitating the implementation of improvement actions based on the results of periodic analyses. 

## 2. Methods

Up to the year 2000, three management plans were defined for these patients:

1. First Plan defined in 1978 [6] (starting point): “*Candida* in the ICU” project.

Problem: *Candida* in non-significant samples and cellular immunity. Patients: Non-neutropenic ICU patients, on mechanical ventilation for >48 h, with previously defined specific conditions (decompensated chronic obstructive pulmonary disease, extracorporeal surgery with complicated postoperative course, abdominal surgery with complicated postoperative course, and patients with severe tetanus). Antifungal therapy: Administered solely when candidemia, endophthalmitis, or positive specimens from sterile sites are identified. Case–control study. The control group consisted of volunteer medical students from the Faculty of Medicine. The cases included in the study comprised selected patients from whom samples were systematically collected every five days from multiple anatomical sites, including pharyngeal swabs, gastric aspirates, bronchial secretions, urine, vaginal swabs, wounds or drains, as well as blood cultures and ophthalmological examinations. Direct microscopic examination was performed according to the type of sample, together with specific cultures for *Candida*. In positive cases, species identification was carried out using standard techniques, including the germ tube test and the ID 32 C system. These methodologies were maintained in subsequent protocols [8].

2. Second Plan defined in 1986 [9]: “*Candida* in the ICU” project.

Problem: High mortality associated with the presence of two or more infectious foci and its correlation with impaired cellular immunity. Patients: Non-neutropenic ICU patients requiring mechanical ventilation for more than 48 h, regardless of underlying pathology. All patients in whom *Candida* was isolated from any sample obtained during the diagnostic work-up of a suspected infection were included. This involved the systematic collection of screening specimens, including pharyngeal swabs, gastric aspirates, bronchial secretions, samples from wounds or drains, blood cultures, and ophthalmological examination findings. Sampling was repeated on a weekly basis as part of a follow-up protocol. Antifungal treatment: Administered according to a predefined therapeutic algorithm, in which multifocal candidiasis (MC) was considered a form of invasive candidiasis (IC). Study design: Prospective cohort study with post-mortem analyses [10]. Patients with *Candida* isolated from a single screening site (defined as *Candida* colonization [CC]; control group) were compared with those presenting with multifocal candidiasis/invasive candidiasis (MC/IC; intervention group).

3. Third Plan defined in 1998 [11]: “*Candida* in the ICU” project.

Problem: Attributable mortality of 25%. The study aimed to determine whether this outcome was related to delayed diagnosis or inadequate antifungal treatment. Patients: Non-neutropenic ICU patients with multiple organ dysfunction syndrome (MODS). All patients in whom *Candida* was isolated from any sample obtained during the diagnostic evaluation of a suspected infection were included. From this stage onward, screening and follow-up procedures were performed in accordance with the protocol established in the second plan. Antifungal treatment: Administered according to a predefined therapeutic algorithm that incorporated clinical severity, evidence of *Candida* infection with potential antifungal resistance, and the presence of severe dysfunction in key organs, particularly the liver and kidneys. Study design: Prospective cohort study with post-mortem analyses. Patients with *Candida* isolated from a single screening site (defined as *Candida* colonization [CC]; control group) were compared with those presenting with multifocal candidiasis/invasive candidiasis (MC/IC; intervention group). Antifungal susceptibility testing was incorporated into the microbiological diagnostic procedures.

The results of the statistical analyses performed at each phase of the Deming Cycle, together with an extensive review of the literature available at the time, informed the improvement strategies incorporated into subsequent action plans. In the final phase, a comparative statistical analysis was conducted between the third plan (intervention group) and the second plan (historical control group). Statistical analyses were performed using SPSS software (version 15.0, SPSS Statistical Program, Hispanoportuguesa SPSS, S.L., Madrid), corresponding to the version available during the study period. Continuous variables were analysed using Student’s *t* test or the Mann–Whitney *U* test, as appropriate, and are presented as mean (standard deviation). Categorical variables were compared using the chi-square test or Fisher’s exact test, as appropriate. Univariate logistic regression analysis was performed to evaluate potential risk factors, with estimation of odds ratios (ORs) and 95% confidence intervals (CIs). Multivariate models were fitted using the BMDP statistical package. All *p*-values were two-tailed, and statistical significance was set at *p* < 0.05.

Ethical statement: Although the data from the different studies were collected prior to the most recent revisions of the Declaration of Helsinki (all studies were conducted in accordance with the ethical principles of the Declaration of Helsinki as established at the 18th World Medical Assembly (Helsinki, Finland, June 1964) [12]. The research methodology was reviewed and approved by the Joint Commission in 1999.

## 3. Results

### 3.1. Candidiasis in the ICU: An Opportunity to Improve Patient Safety?

In the late 1970s, concern regarding nosocomial infections in the ICU was primarily focused on the emergence of infections caused by new species and multidrug-resistant bacterial strains. In this context, non-bacterial infections largely went unrecognized. Candidemia was only occasionally identified, typically in patients receiving parenteral nutrition. In such cases, management was relatively straightforward: removal of the intravascular catheter followed by culture of the catheter tip using the Maki technique [13]. When the catheter tip culture yielded *Candida*, the episode was generally considered resolved, as evidenced by defervescence and negative follow-up blood cultures. In contrast, when the catheter tip culture was negative, uncertainty arose regarding the need to initiate antifungal therapy with amphotericin B, which at the time was the only available agent. In many cases, the clinical outcome was fatal. It remained unclear whether mortality was attributable to *Candida* infection or to the toxicity of antifungal treatment, particularly in critically ill patients who frequently presented with renal impairment related to their underlying condition. A study on candidemia published in 1985 [14] reported no statistically significant differences in mortality between treated (60.71%) and untreated patients (56.41%). These findings raised important questions as to whether mortality among treated patients was related to adverse effects of antifungal therapy or to delays in the initiation of appropriate treatment.

M. Ho (1981) [15] described the challenges of diagnosing *Candida* infection in non-neutropenic critically ill patients, noting that signs of dissemination may be absent or appear too late to be clinically useful. Positive blood or urine cultures are observed in fewer than half of cases, and candidemia may only become evident in the days preceding death. Moreover, even when present, positive blood or urine cultures alone provide insufficient evidence for a definitive diagnosis of disseminated infection. Nevertheless, clinical suspicion should be heightened in the presence of relevant risk factors. In contrast, in leukemic patients with neutropenia, a positive blood or urine culture may be sufficient to support a presumptive diagnosis. Four decades later, these observations remain largely unchanged. The clinical manifestations of invasive candidiasis continue to be non-specific, diagnosis remains highly challenging, and delays in the initiation of antifungal therapy persist, adversely affecting morbidity, length of ICU stay, and associated mortality [16]. Despite advances in antifungal therapy, no substantial reduction in mortality has been consistently demonstrated [17,18,19]. In light of these findings, candidemia should not be considered the gold standard for determining the need for antifungal therapy in ICU patients. However, its continued use as a reference standard is reflected in the fact that the majority of published studies on candidiasis focus primarily on candidemia. The most common sources of candidemia in hospitalized patients include the gastrointestinal tract, representing an endogenous origin associated with broad-spectrum antibiotic use and bacterial translocation, as well as the skin in critically ill patients with indwelling vascular catheters [20,21]. Furthermore, multifocal *Candida* colonization has been identified as an independent risk factor for subsequent invasive infection [8,22,23]. The risk management analysis presented in this document focuses on endogenous *Candida* infection in critically ill non-neutropenic patients, with a primary objective of achieving significant improvements in clinical outcomes. Effective identification of the target patient population is essential, as opportunities for improvement cannot be recognized without it. *Candida* should be conceptualized as an indicator of systemic imbalance, reflecting the combined influence of multiple factors, including the patient’s microbiota, immune status, and hospital environment. It serves as a clinical alert in the context of cumulative, rather than isolated, risk factors, emphasizing the need for a proactive approach in response to clinical suspicion rather than a purely reactive response to positive cultures. From this perspective, *Candida* infection in long-stay ICU patients may function as a sentinel marker, providing insight into the quality of care within a patient safety framework. This approach reflects a shift in understanding: *Candida* is no longer regarded solely as an opportunistic pathogen or secondary problem but as a potential indicator of systemic dysregulation. Critically ill neutropenic patients and cases of exogenous candidiasis were excluded from this review.

### 3.2. Identification of the Target Patient

To identify the target population, neutropenic patients, oncology patients undergoing chemo-radiotherapy, and transplant patients were excluded. At the same time, patients with acquired candidiasis prior to their ICU admission were not included in the study. The target population is characterized by the presence of four criteria, within this cumulative environment of risk factors:

(a) Long ICU stays with mechanical ventilation. Initial observations identified the potential target population as long-stay ICU patients requiring more than one week of mechanical ventilation due to Multiple Systems Organ Failure (MSOF) [24].
(b) Multiple organ dysfunction syndrome. There are long-stay ICU patients receiving invasive therapies for more than a week, in whom the pathology responsible for these therapies is multiple organ failure. In the 1980s, the term MOF [25] was consolidated, associating it with the systemic inflammatory response. However, it was not until the 1990s that the term MODS [26] appeared, incorporating the concept of the potential reversibility of the lesions responsible for the critical state. In 1996, the SOFA score [27,28,29] was developed, a quantitative indicator of the degree of multiple organ dysfunction.
(c) Immunoparalysis. Mortality in ICU patients with MODS is related to the presence of profound immunosuppression [30], as we observed in our studies in the late 1970s. It is not until the 2000–2010 period that the cellular and molecular mechanisms of this immunosuppression are documented. Since 2012, a new concept has been proposed —PICS, or Persistent Inflammatory–Immunosuppression–Catabolism Syndrome— to explain the phenotype of critically ill patients [31]. It is the conceptual evolution from SIRS, through CARS, to the so-called immunoparalysis. It is this idea of immunoparalysis that is integrated into the 2016 Sepsis-3 mode [32]. Currently, a non-neutropenic ICU patient at high risk for disseminated endogenous candidiasis can be defined as a patient with a prolonged ICU stay, intubated and receiving mechanical ventilation for more than one week, treated with broad-spectrum antibiotic therapy, and who is affected by immunoparalysis secondary to MODS and a SOFA ≥ 5.
(d) *Candida* isolation and superinfection. Without identifying the presence of *Candida* in the patient, the risk of candidiasis cannot be established.

Given the potential direct relationship between candidiasis and cellular immune dysfunction—and considering that cellular immune deficits had been previously described, particularly in critically injured polytrauma patients—it was decided in the late 1970s to systematically study the presence of *Candida* in various body fluids according to standardized criteria. Concurrently, in vivo and in vitro assessments of cellular immunity were performed within the first 48 h of ICU admission. The results demonstrated that patients with the cellular immune deficiency associated with multifocal candidiasis, or those at high risk of disseminated candidiasis, exhibited significantly higher mortality (17/23; 74%) compared to patients in the colonization group (single positive sample), who had a mortality of 6/20 (30%) (χ^2^ = 9.41, *p* < 0.016 [6]).

### 3.3. Candidiasis: Terminology

*Candida albicans* is the most prevalent saprophyte in the human upper and lower gastrointestinal tract as well as the female genital tract. In a study of 86 healthy adults, oropharyngeal colonization was observed in 30% of subjects, while the stomach remained free of infection; however, 35–65% of samples from the small intestine and colon were positive for *Candida* [33]. In patients with AIDS, a correlation has been reported between oral candidiasis and *Candida* esophagitis [5]. These observations align with our findings regarding the definition of the abdominal focus. Notably, the development of *Candida* esophagitis was often asymptomatic due to both the patient’s critical condition and ongoing treatments. A positive pharyngeal swab alone was not sufficient to classify this site as part of a multifocal infection. However, simultaneous positivity of a pharyngeal swab and gastric aspirate obtained via a nasogastric tube was strongly predictive of developing *Candida* esophagitis, a finding confirmed in initial post-mortem studies. The concurrent positivity of these two samples defined the gastrointestinal focus. Multifocal infection was further defined by:

- Respiratory focus: Bronchial samples obtained via endotracheal tube (aspirate or brush);
- Urinary focus: Sterile catheter samples;
- Other foci: Wound exudates or surgical drains.

The genital focus in women was excluded from the analysis due to the high prevalence of *Candida* as part of the normal saprophytic flora, particularly among diabetic women.

Once a standard for the foci defining multifocality was established, the terminology was agreed upon (Table 1):

- *Candida* colonization (CC): Isolation of *Candida* of the same species in a single focus, or in more than one focus in a patient without evidence of MODS with immunoparalysis (SOFA < 5). These patients do not require empiric antifungal treatment or prophylaxis. At most, they may require monitoring for *Candida* if the ICU stay is prolonged.
- Candidiasis: *Candida* infection in a patient with evidence of MODS and immunoparalysis (SOFA ≥ 5). Patients in this group all require antifungal treatment as soon as possible. This group can be further differentiated into:
- Disseminated candidiasis (DC): When there is evidence of dissemination, either through an ophthalmologic study (endophthalmitis), or from pathological fluid samples obtained from a closed cavity (abscess, CSF, pleural, pericardial or peritoneal), or from histopathological studies of biopsy samples. Many authors equate this concept with invasive candidiasis.
- Invasive candidiasis (IC): This is simply evidence of fungal infiltration beyond the submucosa and the demonstration of regional vascular invasion. It can be in the lungs, genitourinary tract, oesophageal/digestive tract, or in contaminated wounds. This is the major step for dissemination. Its diagnosis requires a biopsy and histopathological examination.
- Multifocal candidiasis (MC): defined as the simultaneous isolation of *Candida* in two or more sites, according to previously defined criteria. Although it does not objectively demonstrate pathological invasion, this multifocal nature represents dissemination throughout all mucosal surfaces. If this extension occurs in a patient with immunoparalysis, there is a high risk of multifocal invasive candidiasis. To speak of multifocal candidiasis, the presence of two or more foci must be demonstrated in a patient with MODS and immunoparalysis (SOFA ≥ 5). This is why multifocal candidiasis was equated with invasive candidiasis. Both cases are patients who can be considered at high risk for disseminated candidiasis.
- Candidemia: it is nothing more than the isolation of *Candida* in the bloodstream, through blood cultures. It has two forms. The most common is secondary to catheter contamination, usually parenteral nutrition catheters. In this case, the problem is resolved by removing the catheter and confirming contamination of its tip. This is the only situation where one might consider avoiding antifungal treatment. If this is not the case, candidemia is usually a very late sign that the patient is suffering from disseminated candidiasis. In this situation, antifungal treatment is often delayed.

### 3.4. Standardisation and Decision-Making Algorithm

**Figure 2:**
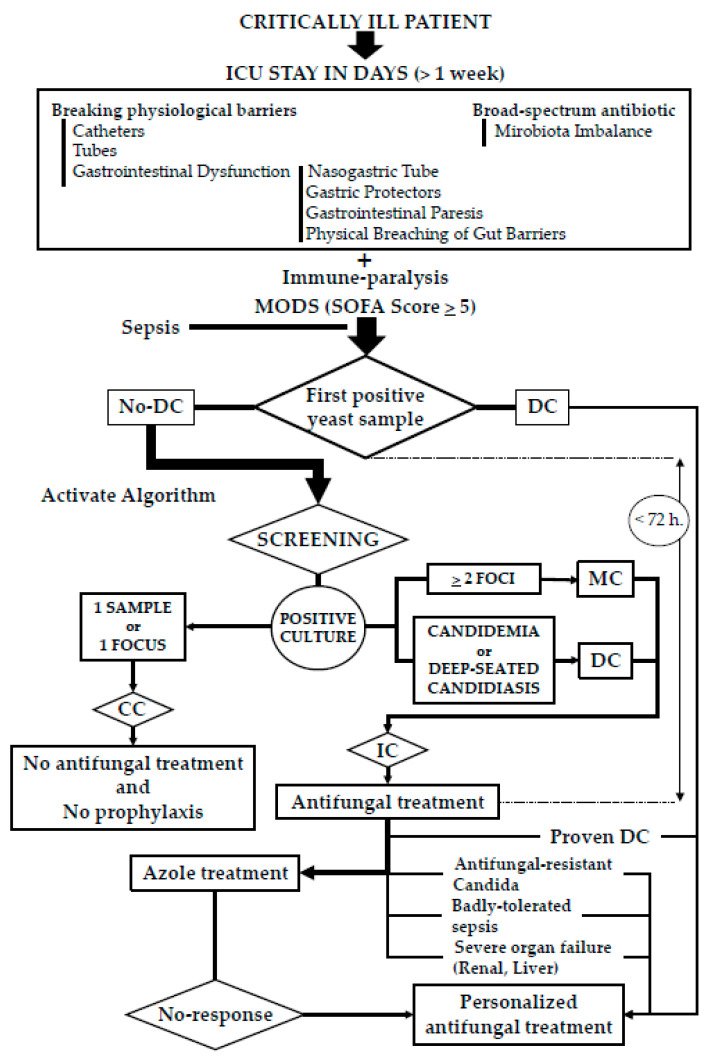
Diagnostic–therapeutic algorithm defined after completion of the third Deming cycle. The starting point is a non-neutropenic ICU patient with a stay exceeding one week, affected by multiple organ dysfunction syndrome (MODS), and a SOFA score > 5. During the ICU stay, if clinical signs of superinfection develop, routine investigations are performed to identify the causative pathogen and the source of infection. If *Candida* is detected in any routine culture sample, specimens are immediately collected from multiple sites according to protocol to perform *Candida*-specific studies. Results from these analyses should be available within 72 h to enable the timely application of the therapeutic algorithm and to avoid delays in initiating antifungal therapy when indicated. Definitions: DC: Disseminated candidiasis. MC: Multifocal candidiasis. IC: Invasive candidiasis. CC: *Candida* colonization. Sampling sites: Respiratory: bronchial secretions. Digestive: gastric aspirate and pharyngeal swab. Urinary: urine. Other sites: surgical drains and wounds.

To date, the diagnosis of *Candida* infection has relied primarily on culture results, which necessitate a 48-hour waiting period before initiating antifungal therapy. Although other laboratory tests provide valuable information [34], they have not yet been validated for guiding the early initiation of antifungal treatment. Consequently, these serological assays have not been incorporated into the diagnostic algorithm. The diagnostic and therapeutic algorithm presented here is the culmination of studies conducted between 1978 and 2002 (Figure 2). Recognizing that *Candida* infection represents an ICU-acquired condition, the algorithm was originally developed in the late 1970s as a strategy to enhance the safety of long-stay ICU patients. Over time, it has been refined in accordance with the findings of successive studies, following the PDCA (Plan, Do, Check, Act) or Deming Cycle model, with the overarching goal of promoting continuous quality improvement [35].

The diagnostic component of the algorithm has undergone a few modifications, primarily refining the concept of immunoparalysis in target patients by linking it to the SOFA score. In contrast, the therapeutic component has been modified more extensively, reflecting both the introduction of new generations of antifungal agents and the need for greater individualization of treatment. Individualized therapy takes into account the identification of resistant yeast species, such as *Candida glabrata*, whether the initial sample indicates disseminated candidiasis or detects candidemia not attributable to catheter contamination, the presence of a critical septic state, and the severity of organ dysfunction, particularly involving the kidneys and liver.

### 3.5. Trends in Results Following the Implementation of the Deming Cycle

The first study, conducted at Hospital del Mar in Barcelona (Spain) between 1980 and 1981, included 43 patients selected according to the previously described criteria. Immune response parameters were compared with a control group of healthy subjects. In parallel, these patients were followed every five days, with samples collected from multiple sites as previously described, to monitor *Candida* colonization. Among the 43 patients, 20 (46.5%) were classified as *Candida* colonization (CC) and 23 (53.5%) as multifocal candidiasis (MC). A higher proportion of MC was observed among patients with ICU stays exceeding 12 days (10/20 [50%] in the CC group vs. 21/23 [91%] in the MC group; χ^2^ = 9.07, *p* = 0.0026). Mortality was significantly higher in the MC group (17/23; 74%) compared to the CC group (6/20; 30%) (χ^2^ = 8.29, *p* = 0.004), yielding an ICU mortality ratio (MC/CC) of 2.47. Among patients with ICU stays > 12 days, mortality remained markedly higher in MC cases (15/21; 71%) versus CC cases (1/10; 10%) (χ^2^ = 10.24, *p* = 0.0014). Altered skin immunity tests at ICU admission were more frequent in MC patients with prolonged stays (12/21; 57%) compared to CC patients (1/8; 13%) (χ^2^ = 6.25, *p* = 0.007). Significant differences were also observed in lymphoblastic transformation test (LTT) results obtained at 10–12 days of ICU stay, with survivors showing higher LTT values than non-survivors (37 vs. 20.78; *t* = 3.76, *p* = 0.002). The LTT results in the healthy control group were 39.25.

The subsequent two studies, conducted between 1988 and 1995 and between 2000 and 2002 at the Hospital General de Catalunya, Barcelona, Spain, further explored *Candida* infection in critically ill patients. The publication of preliminary results from the second study [9] prompted the organization of a Consensus Conference on *Candida spp.* in critically ill patients in Spain (1999) [36], as well as the initiation of a subsequent multicenter study, EPCAN [37]. Findings from the EPCAN study contributed to the development of the *Candida* Score, a scoring system analogous to the *Candida* Colonization Index [38]. However, these scoring systems were not incorporated into the diagnostic algorithm of the third study. Table 2 summarizes the characteristics of the selected patient populations, comparing the study conducted between 1988 and 1995 [8,39] with that from between 2000 and 2002 [11].

The study conducted between 1988 and 1995 [39] incorporated the concept of attributable mortality into its results analysis [40,41]. It demonstrated that, in ICU patients with endogenous candidiasis, the EORTC Consensus [42] recommendations for the management of suspected candidiasis are not directly applicable. Applying the concept of treating multifocal candidiasis, attributable mortality was 25%. The series with the most favorable outcomes reported an attributable mortality of 26% [43]. In our cohort, 2.5% of patients developed endophthalmitis, and 15% experienced candidemia. By comparison, a study published in The Lancet (2003) reported rates of 3.7–25% for endophthalmitis and 10–20% for candidemia [44]. The hospital crude mortality rate ratio between patients with candidiasis and those with *Candida* colonization was 2.12 (50.8% vs. 24%; *p* < 0.05).

In the study conducted between 2000 and 2002 [11,45], the attributable mortality of *Candida* infection was markedly reduced, reaching 4.8%. No cases of endophthalmitis were detected, and candidemia was identified in only 3% of patients. The hospital crude mortality rate ratio between patients with candidiasis and those with *Candida* colonization was 0.92, a difference that was not statistically significant (NSD). Table 3 presents a comparison of mortality between the two study periods: 1988–1995 versus 2000–2002.

## 4. Discussion

The reference standard for diagnosing invasive candidiasis (IC) remains culture, particularly from sterile sites such as blood, peritoneal fluid, or pleural fluid. Although blood cultures are still considered the gold standard, their sensitivity is low, identifying only approximately 50% of patients with IC. This limitation has prompted the search for rapid diagnostic tests that do not depend on culture results [46]. The role of non-culture-based diagnostic tests in the diagnosis and management of candidiasis remains unclear. These assays are generally faster and more sensitive than blood cultures; however, they cannot provide species identification or antifungal susceptibility [4,47]. T2 magnetic resonance (T2MR) has emerged as a promising diagnostic tool for candidemia, demonstrating a sensitivity of 91.1% and specificity of 99.4%, with a turnaround time of approximately three hours. In addition, T2MR allows targeted antifungal therapy by identifying key *Candida* species, including *C. albicans*, *C. tropicalis*, *C. parapsilosis*, *C. krusei*, and *C. glabrata* [16,48,49]. However, these studies have been limited to patients with suspected candidemia. Further research is needed to evaluate the performance of T2MR in cases of invasive candidiasis without candidemia [49,50]. To date, studies continue to support positive blood cultures as the gold standard [49], and most research remains based on this criterion. It would be valuable to include ICU patients with multifocal candidiasis (MC) and *Candida* colonization (CC), as previously defined, to assess the sensitivity and specificity of T2MR in these populations. Simultaneously, this approach would allow evaluation of whether these tests could improve antifungal management [51]. Advanced non-culture-based technologies—including PCR, T2*Candida*, microfluidic chips, next-generation biosensors, nanotechnology, and artificial intelligence–based models—aim to reduce diagnostic time and enable earlier initiation of antifungal therapy [52]. The overarching goal is to identify high-risk patients for candidiasis, facilitating not merely empirical treatment but early treatment guided by diagnostic evidence. Despite the development of novel antifungal agents and microbiological techniques, mortality rates for invasive candidiasis (IC) in non-neutropenic ICU patients have not improved substantially over recent decades [19,53]. Crude ICU mortality ranges from 28% to 50% [53,54,55,56,57], while attributable mortality has been estimated between 15% and 50% [18,58,59,60]. The introduction of echinocandins has not resulted in significant reductions in attributable mortality [61]. The evolution of candidiasis management using a methodology focused on patient safety has enabled a significant reduction in crude mortality to 25% and attributable mortality to 5% (with an IC/CC hospital mortality ratio of 0.99), as well as a reduction in candidemia to 5% and the elimination of endophthalmitis cases. Limitations of the study include the inability to evaluate emerging advanced non-culture-based diagnostic technologies or newer generations of antifungal agents. Furthermore, the impact of emerging resistant *Candida* species, such as *C. auris*, could not be assessed. No significant changes were observed over time in the clinical characteristics of ICU patients who are at high risk for candidiasis. An extensive literature review revealed that the patients included in this series share clinical characteristics consistent with those reported in other studies.

## 5. Conclusions

The introduction of a surveillance system and strategic guideline for candidiasis in non-neutropenic patients with prolonged ICU stays, MODS, and SOFA > 5 has made it possible to improve patient outcomes. In our series, not only were crude mortality and attributable mortality reduced, but there was also a significant reduction in candidemia, endophthalmitis, and evidence of candidiasis in post-mortem studies. In conclusion, *Candida spp.* in the ICU may serve as a biomarker of patient fragility, reflecting complex systemic imbalances. This perspective highlights the importance of balancing proactive clinical management with caution against indiscriminate antifungal overuse and an exclusive reliance on laboratory test results.

## 6. Future Directions

### Improvement Objectives

If the vision for continuous improvement is maintained, several additional areas for advancement can be identified:

1. Monitoring antifungal resistance: Identification and surveillance of emerging strains resistant to commonly used antifungals, as well as the appearance of new species such as *Candida auris* [62].
2. Reduction in treatment-related adverse effects: Strategies to minimize nephrotoxicity, hepatotoxicity, and other complications associated with antifungal therapy.
3. Validation of advanced diagnostics: Confirming the clinical relevance of non-culture-based technologies to enable their integration into diagnostic algorithms [52].
4. Cost control: Optimization of healthcare resources in the management of ICU patients at risk for candidiasis.
5. Immunoparalysis monitoring: Development of biomarkers to monitor immunoparalysis in patients with MODS, as recently proposed [63,64,65,66]. Given the complexity of this syndrome, it may be necessary to develop a composite score, analogous to the SOFA score, to define the severity of MODS. The SOFA and qSOFA [67] scores have proven extremely useful for stratifying severity during the COVID-19 pandemic, and a similar pragmatic strategy could provide clinicians with a clear assessment of patients’ immune status [68]. Existing proposals, such as the REALISM [69] or REALIST scores [68], may offer a feasible path forward.
6. Therapeutic implications: Combining a SOFA-derived immunoparalysis score with existing severity assessments could guide interventions to prevent endogenous candidiasis and other opportunistic ICU infections, potentially improving survival, reducing prolonged ICU stays, and lowering associated healthcare costs.
7. Early detection and targeted therapy: Timely identification of immunoparalysis is critical for determining whether interventions targeting this condition should be initiated and for predicting the risk of *Candida* invasion. Therapeutic strategies aimed at reversing or mitigating immunoparalysis in this patient population should also be considered.

In conclusion, *Candida* in the ICU may serve as a biomarker of patient fragility, reflecting complex systemic imbalances. This perspective highlights the importance of balancing proactive clinical management with caution against indiscriminate antifungal overuse and an exclusive reliance on laboratory test results.

## Figures and Tables

**Table 1:** Definition of *Candida* colonization (CC), *Candida* colonization (CC) refers to the presence of the yeast in one or more non-sterile sites in long-stay ICU patients, particularly those with multiple organ dysfunction syndrome (MODS). CC does not require prophylactic or empirical antifungal therapy but mandates systematic monitoring. In contrast, candidiasis or *Candida* infection necessitates prompt initiation of antifungal treatment, tailored to the patient’s clinical condition, the risk of drug-related nephrotoxicity or hepatotoxicity, and the potential presence of multidrug-resistant *Candida* species. Candidiasis encompasses several entities, including disseminated candidiasis (DC), multifocal candidiasis (MC)—considered equivalent to invasive candidiasis (IC)—and candidemia. While DC and candidemia may be identified through cultures obtained under clinical suspicion of sepsis, CC and MC require standardized screening protocols to determine the number and distribution of affected sites. These include the gastrointestinal tract, respiratory tract, urinary tract, and “other” sites, referring to specimens obtained from wounds or surgical drains.

		1st. Sample/Screening	Evidence of MODS SOFA ≥ 5
***Candida* Colonization (CC)** **No profilaxis/No treatment**		1 sample +/1 focus + Non-sterile site	No or Yes
Candidiasis (*Candida* Infection) *Antifungal treatment*	Disseminated Candidiasis (DC)	Sample of sterile cavities, endophalmitis, histopathological study	Yes or No
	Multifocal Candidiasis (MC)	1 sample +/2–4 foci + Non-sterile site	Yes or No
	Candidemia	Bloodstream positive	Yes or No

**Table 2:** Comparison of demographic data of patients included in 1988–1995 versus 2000–2002, and differences between the two groups regarding disease severity and treatments administered in patients with candidiasis. SD = standard deviation; OR = odds ratio; CI = confidence interval.

	1988–1995		2000–2002				
	Control Group		Intervention Group				
	*n*	% or SD	*n*	% or SD	OR Adjust	95% CI	*p*-Value
Candidiasis/Total ICU patients prevalence	120/3389	4%	60/1904	3%			
Candidiasis/Total *Candida* isolation	120/145	83%	60/102	59%			
Disseminated Candidiasis	31/120	26%	4/60	7%	0.28	0.09 to 0.87	0.028
Candidemia	18/120	15%	3/60	5%	0.27	0.07 to 1.06	0.06
Endophthalmitis	3/120	3%	0/60				
Gender (Males/total *Candida* isolation)	111/145	77%	51/102	50%			<0.01
Mean age	53	22	62	20			<0.01
Surgical patients	85/145	59	32/102	31%			<0.01
APACHE III first-day ICU Candidiasis	78	26	81	25			
SOFA first positive culture *Candida* Candidiasis	9	3	9	3			
Length of stay in ICU Candidiasis	32	18	26	20			0.03
Fluconazole Treatment/Candidiasis	84/120	70%	54/60	90%			<0.01
Amphotericin’s Treatment/Candidiasis	42/120	35%	13/60	22%			
Fluconazole-resistant yeast (*C. glabrata* or *C. krusei*)	17/145	18%	10/102	12%			0.68
Hepatotoxicity (Fluconazole)			3/54	6%			
Nephrotoxicity (Amphotericine-B)	4/37	11%	3/13	23%			

**Table 3:** Comparison of mortality among patients included in 1988–1995 versus 2000–2002. SD = standard deviation; OR = odds ratio; CI = confidence interval.

	1988–1995		2000–2002				
	Control Group		Intervention Group				
	*n*	% or SD	*n*	% or SD	OR Adjust	95% CI	*p*-Value
ICU Mortality Candidiasis	46/120	38%	15/60	25%	0.37	0.18 to 0.80	0.011
Hospital Mortality Candidiasis	61/120	51%	21/60	35%	0.37	0.17 to 0.79	0.01
Statistical Attributable Mortality	17/61	28%	1/21	5%	0.10	0.012 to 0.83	0.033
Post-Mortem Attributable Mortality	10/36	28%	0/8	0%	−0.27	−0.42 to −0.13	
ICU Mortality Ratio (Candidiasis/CC)	1.9		0.81				
Hospital Mortality Ratio (Candidiasis/CC)	2.12		0.99				0.016

## Data Availability

The original contributions presented in this study are included in the article. Further inquiries can be directed to the corresponding author.
